# Silver-Russell syndrome: genetic basis and molecular genetic testing

**DOI:** 10.1186/1750-1172-5-19

**Published:** 2010-06-23

**Authors:** Thomas Eggermann, Matthias Begemann, Gerhard Binder, Sabrina Spengler

**Affiliations:** 1 RWTH Aachen, Institute of Human Genetics, Aachen, Germany; 2 University of Tübingen, Children's Hospital, Tübingen, Germany

## Abstract

Imprinted genes with a parent-of-origin specific expression are involved in various aspects of growth that are rooted in the prenatal period. Therefore it is predictable that many of the so far known congenital imprinting disorders (IDs) are clinically characterised by growth disturbances. A noteable imprinting disorder is Silver-Russell syndrome (SRS), a congenital disease characterised by intrauterine and postnatal growth retardation, relative macrocephaly, a typical triangular face, asymmetry and further less characteristic features. However, the clinical spectrum is broad and the clinical diagnosis often subjective. Genetic and epigenetic disturbances can meanwhile be detected in approximately 50% of patients with typical SRS features. Nearly one tenth of patients carry a maternal uniparental disomy of chromosome 7 (UPD(7)mat), more than 38% show a hypomethylation in the imprinting control region 1 in 11p15. More than 1% of patients show (sub)microscopic chromosomal aberrations. Interestingly, in ~7% of 11p15 hypomethylation carriers, demethylation of other imprinted loci can be detected. Clinically, these patients do not differ from those with isolated 11p15 hypomethylation whereas the UPD(7)mat patients generally show a milder phenotype. However, an unambiguous (epi)genotype-phenotype correlation can not be delineated.

We therefore suggest a diagnostic algorithm focused on the 11p15 hypomethylation, UPD(7)mat and cryptic chromosomal imbalances for patients with typical SRS phenotype, but also with milder clinical signs only reminiscent for the disease.

## Review

Over the past 20 years it has become increasingly clear that genomic imprinting is of great relevance for human diseases. Imprinted genes with a parent-of-origin specific expression are involved in various aspects of growth and behaviour that are rooted in the prenatal period and indeed, many of the so far known congenital imprinting disorders (IDs) are clinically characterised by growth disturbances. While Angelman, Prader-Willi and Beckwith-Wiedemann syndromes (BWS) are well established IDs, imprinting defects in patients with Silver-Russell syndrome (SRS) are relatively new findings.

The main features of SRS [RSS; OMIM 180860] are severe intrauterine and postnatal growth retardation, relative macrocephaly and a characteristic small, triangular face. The disease is associated with additional dysmorphic features including fifth finger clinodactyly and hemihypoplasia (table 1). Although a clinical scoring system to assist the diagnosis has recently been suggested [1], the accuracy of diagnosis is influenced by the experience of the clinical investigator. Furthermore, the clinical picture of SRS in adulthood is less clear than in early childhood.

**Table 1:** Clinical features in SRS and their frequencies in the different molecular subgroups.(*combined from different studies by [59])

clinical features	**total RSS n = 143 **[58]	**UPD(7)mat **[59]	**ICR1 hypomethylation carriers **[59]	**idiopathic RSS **[59]
*growth parameters*				n = 129
birth weight (SD)	94% (< 3 Perc.)	-2.79 (n = 37)	-3.55 SD (n = 58)	-3.1

birth length (SD)		- 3.1 (n = 32)	-4.38 SD (n = 57)	-4.1

birth OFC (SD)		- 1.26 (n = 28)	-1.35 SD (n = 54)	-1.5

postnatal growth retardation (SD)	99%	-3.37 (n = 38)	-3.41 (n = 49)	-3.6

*clinical features*				n = 388
relative macrocephaly	64%	92%	91% (n = 59)	68.4%

muscular hypotonia	45%	69.2% (n = 13)	-	45%

asymmetry	51%	60% (n = 30)	77% (n = 57)	53.1%

clinodactyly V	68%	82% (n = 34)	78% (n = 40)	69.9%

squeaky voice	22%			

developmental delay	37%	43% (n = 39)	20.5% (n = 31)	32.2%

*craniofacial features* triangular face	79%	97% (n = 34)	76% (n = 59)	78.4%
prominent forehead		68%	88% (n = 34)	72.4%

downturned corners of the mouth	46%	50% (n = 22)	55% (n = 9)	57.3%

micrognathia		73% (n = 15)	55% (n = 9)	44%

ear anomalies	53%	78.6% (n = 14)		40.3%

teeth anomalies	28%	64% (n = 14)	0% (n = 7)	28%

The frequency of SRS is currently unknown, but it is probably underdiagnosed due to the broad range of features [1].

The influence of genetic factors in the aetiology of SRS is documented by classical genetic findings such as familial cases of SRS and cytogenetic aberrations. Most cases of SRS are sporadic, but familial cases have been reported (for review: [2]). Duncan et al. [2] suggested that most familial cases were transmitted in an autosomal dominant manner with marked intrafamilial variability. An autosomal recessive inheritance has been supposed in eight families but in six of them the clinical documentation is questionable (for review: [3]). However, in only two families a recurrence of (epi)mutations in 11p15 have been described [4].

In the following, an overview on the currently known (epi)genetic disturbances in SRS will be given. We want to emphasize that the order of mutations does not reflect the importance but their chronological identification.

## Chromosomal aberrations and SRS

Several SRS patients have structural aberrations affecting numerous chromosomes, but only chromosomes 7, 11, and 17 were consistently involved in individuals fulfilling strict diagnostic criteria of SRS. Based on balanced translocations in two patients involving 17q24-q25 [5,6], a central role of this chromosomal region in SRS aetiology had been discussed for a long time. However, characterisation of the 17q breakpoints in both patients showed that they were not identical [7]. The reported heterozygous deletions in the growth hormone (*GH*) gene cluster in 17q [8] are now regarded as apathogenic polymorphisms [9].

However, conventional cytogenetic analysis was hampered in the past by the low microscopic resolution. Thus the development of array-based techniques for molecular karyotyping now allows the identification of cryptic imbalances which formerly escaped microscopic analysis. Meanwhile two studies reported on SRS patients carrying small deletions/duplications < 3 Mb [10,11]. In respect to genetic counselling, conventional karyotyping should be considered in the patients' parents to detect balanced rearrangements.

## Chromosome 7 and SRS

Cytogenetic aberrations of chromosome 7 including duplications of 7p11.2p13 and small marker chromosomes have been identified in several SRS individuals (for review: [12,13]). The first evidence for an involvement of this chromosome in the aetiology of the disease was based on the identification of maternal uniparental disomy of chromosome 7 (UPD(7)mat) in ~10% of SRS individuals [14](table 1). Microsatellite typing patterns in nearly all these UPD(7)mat carriers were consistent with the mechanism of trisomic rescue. Due to the trisomic origin of UPD(7)mat it might be assumed that trisomy 7 mosaicism is involved in the aetiology of SRS. This hypothesis was corroborated by the observation of a significant increase of the frequency of completely skewed X inactivation in SRS as a marker for undetected trisomy 7 in the patients and/or their placentas [15]. Nevertheless, two studies detected neither trisomy 7 cells in leukocytes nor in fibroblasts of SRS patients [16,17], probably due to the lethality of general trisomy 7 mosaicism and the limited number of tissues analysed.

In cases of UPD, reduction to homozygosity of a recessive allele is a further cause for aberrant phenotypes, and indeed UPD was first described in a growth retarded patient with cystic fibrosis who was homozygous for a *cystic fibrosis transmembrane regulator *gene mutation caused by a UPD(7)mat. Thus, recessive mutations might be regarded as causative for the SRS phenotype in UPD(7)mat patients, but there is not a common isodisomic segment. This finding excludes a "simple" recessive gene responsible for SRS [18].

In conclusion, the most probable explanation for the SRS phenotype in UPD(7)mat carriers is the disturbed expression of imprinted genes on chromosome 7. UPD(7)mat is generally associated with growth retardation and SRS-like features whereas UPD(7)pat is not (for review: [19]). It has therefore been hypothesized that (i) a diminished expression of paternally expressed gene(s) or (ii) an overexpression of maternally expressed factor(s) on chromosome 7 causes SRS.

So far, research on chromosome 7 encoded factors has focused on two chromosomal segments in 7p and 7q, respectively. For the candidate region in 7p11.2-p13 SRS patients with duplications have been reported (for review: [12,20]). The region harbours an imprinted gene (*growth factor receptor bound protein 10/GRB10*) and several factors involved in human growth and development (*IGFBP1; IGFBP3; PHKG1; EGFR; GHRHR*). Pathogenic mutations in these genes have been excluded in SRS [for review: [21]]. In particular, *GRB10 *plays an essential role in growth and is therefore still a good candidate for SRS. This assumption is supported by a recently published family carrying a maternally inherited dup(7)(p11.2p12) not including *GRB10 *and without SRS features [13]. Nevertheless, neither point mutations in the coding region nor aberrant methylation of *GRB10 *have been detected in SRS patients despite extensive screening studies (for review: [22-24]).

On the other hand, there is evidence that the chromosomal region 7q31 is also involved in SRS aetiology: meanwhile four growth retarded patients with segmental UPD(7q) have been identified [25-27]. In 7q31, three imprinted genes (*MEST/PEG1; CPA4; COPG2*) and two imprinted non-coding RNAs (*MESTIT, CIT1/COPG2IT1*) are localised but screening studies did not detect any pathogenic variants [for review: [21]]. Furthermore, isolated imprinting defects as reported for other imprinting disorders have not yet been detected at the *MEST/PEG1 *locus in SRS patients [28].

Because intrauterine growth retardation has also been reported for UPDs of other chromosomes and is often associated with confined placental mosaicism in human pregnancies, several studies investigated the origin of other chromosomes by microsatellite typing in SRS patients. However, further UPDs have not been observed [29-31].

## Chromosome 11p15 and SRS

Currently, the largest molecular genetic subgroup of SRS are individuals with epimutations and mutations in the chromosomal region 11p15. First evidence for an involvement of this region in the aetiology of the disease was the identification of maternal 11p15 duplications in growth retarded patients. Four out of these six cases showed SRS features in addition to intrauterine and postnatal growth retardation (for review: [32]). Interestingly, the opposite disturbance - duplication of paternal 11p15 - is associated with BWS. Numerous genetic and epigenetic alterations can be detected in BWS patients (for review: [33]) but in more than 50% aberrant methylation patterns in 11p15 are involved. The search for epimutations in 11p15 in SRS patients was therefore consequent and indeed, hypomethylation at the telomeric ICR1 in 11p15 regulating *H19 *and *IGF2 *expression could be identified in 38-63% of cases [34-37] (table 2).

**Table 2:** Frequencies of the different types of (epi)mutations in SRS

Type of (Epi)mutation	Frequency in SRS
11p15: ICR1 hypomethylation	~40%
UPD(7)mat	~10%
11p15 duplications of maternal material	1-2%
further chromosomal aberrations	~1%

**Figure 1:**
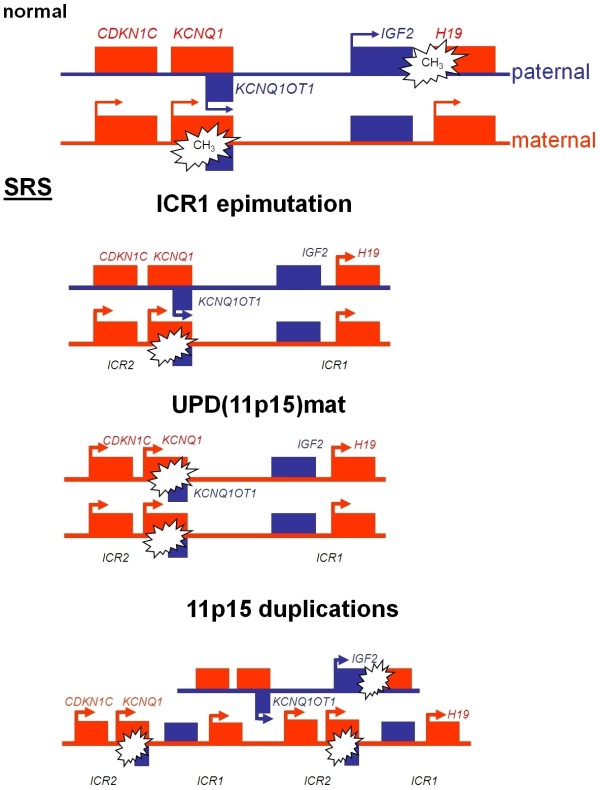
**Epigenetic regulation of the two imprinting centre regions (ICR) in 11p15 and illustration of the types of (epi)mutations detectable in SRS**.

The 11p15 imprinting cluster contains a number of imprinted genes the expression of which is regulated by two different imprinting control regions (ICR1 and ICR2), also called H19 DMR (differentially methylated region) and KvDMR1 (fig. 1) and which are crucial for the control of fetal growth.

The telomeric ICR1 confers a differential chromatin architecture to the two parental alleles leading to reciprocal expression of *H19 *and *IGF2*. The two genes are coexpressed in endoderm- and mesoderm-derived tissues during embryonic development and compete for the same enhancers. The paternally expressed *IGF2 *is involved in fetal development and growth [38,39]. Although *H19 *was one of the first noncoding transcripts identified, its function is still unknown. Knockout of *H19 *removing the whole RNA coding sequence but leaving the promoter and surrounding transcription unit intact had no effect on the imprinted expression of *IGF2 *[40]. These results indicate that the RNA itself might be non-functional, however the fact that *H19 *is a relatively highly conserved gene among mammals (77% identity between human and mouse) suggests a profound functional relevance. A recent study suggests that *H19 *functions as a primary micro RNA precursor involved in the posttranscriptional downregulation of specific mRNAs during vertebrate development [41]. The ICR1 contains seven CTCF target sites (CTCF1-CTCF7) in the differentially methylated region 2 kb upstream of *H19 *and shows allele specific methylation. The zinc-finger binding factor CTCF binds to the maternal unmethylated ICR1 copy and thereby forms a chromatin boundary. This CTCF binding mechanism blocks *IGF2 *expression and promotes *H19 *transcription of the maternal 11p15 copy.

The centromeric ICR2 regulates the (reciprocal) expression of *CDKN1C*, *KCNQ1 *(*potassium channel KQT-family member 1*) and further genes and is methylated only on the maternal allele. Mutations in the paternally suppressed *CDKN1C *gene account for up to 40% of familial BWS cases and 5-10% of sporadic patients (table 1). The gene encodes a cyclin dependent kinase inhibitor (p57^KIP2^) and is part of the p21^CIP2^Cdk inhibitor family. Functional analysis of *CDKN1C *germline mutations detected in two BWS patients showed the loss of cell-cycle inhibition [42]. The gene of another non-coding RNA in 11p15, *KCNQ1OT1 *(*LIT1*), is localised in intron 9 of the *KCNQ1 *gene. *KCNQ1OT1 *is expressed by the paternal allele and probably represses realisation of the *CDKN1C *gene. Loss of methylation (LOM) of the maternal ICR2 allele correlates with expression of *KCNQ1OT1*. In BWS, one central physiological change caused by ICR2 (epi)mutations (hypomethylation at ICR2) as well as *CDKN1C *point mutations is the reduced expression of *CDKN1C*.

The 11p15 epimutation in SRS is typically a hypomethylation of the telomeric ICR1 (table 1). In contrast, the most frequent alteration in BWS is hypomethylation at the centromeric ICR2 accounting for ~50% of patients, whereas ICR1 hypermethylation is diagnosed in only 2-7% of BWS patients. Clinically, the majority of ICR1 hypomethylation carriers fulfil the clinical criteria of SRS [34] but the epimutation has also been diagnosed in patients with only growth retardation and asymmetry [36,43]. However, this disturbance has not yet been detected in individuals with isolated pre- and postnatal growth restriction [44].

The recent identification of a SRS patient with a duplication restricted to the ICR2 [45] suggests that both ICRs on 11p15 are involved in the aetiology of the disease, like in BWS. This finding and further data obtained from BWS patients and from studies in mice suggest that ICR1 and ICR2 interact [46].

## Postfertilisation origin of 11p15 hypomethylation and aberrant methylation at multiple loci in SRS

The mosaic distribution of the 11p15 epimutation in nearly all SRS patients can be attributed to a post-fertilisation error. Clinically, this mocaicism is reflected by hemihypoplasia, which is present in the majority of these patients.

The data from twin studies are consistent with this mosaic distribution of epimutations. In SRS, four discordant but only one concordant monozygotic pair have been observed (for review: [47]). These ambiguous findings are consistent with the data of Gicquel et al. [35]. They reported discordant monozygotic twins carrying the ICR1 epimutation in blood, but the affected twin had LOM in skin fibroblasts. There have been similar observations for the ICR2 locus in discordant BWS twins with the same epigenetic defect in lymphocytes but different methylation patterns in fibroblasts or buccal mucosa [48,49]. In conclusion, the post-fertilisation origin of the ICR1 or ICR2 epimutations explains the high rate of discordance between twins with SRS or BWS.

Further evidence for postzygotic defects in the establishment of imprinting marks is based on observations in patients with transient neonatal diabetes mellitus (TNDM), SRS and BWS who have LOM at further maternally (and paternally) imprinted loci, in addition to methylation defects typical for each disease [50,51]. In cases of TNDM, the patients with additional LOMs had a phenotype that is slightly different from those with TNDM and LOM at 6q24 only, probably caused by the altered methylation at the other imprinted loci. Based on these findings, Mackay and coworkers [52] proposed the existence of a maternal hypomethylation syndrome. Recently, also BWS and SRS patients with multilocus hypomethylation in blood lymphocytes have been reported [49,51,53]. In these patients both paternally and maternally imprinted loci were affected in leukocytes. In monozygotic (MZ) twins discordant for BWS Bliek and coworkers [49] observed similar imprinting anomalies of one or more loci in leukocytes of both twins but in buccal swab DNA the epimutations were detectable only in the affected twin. In all studies, a phenotypic difference between BWS or SRS patients with multiple hypomethylated loci and patients carrying isolated 11p15 epimutations was not obvious.

Summarizing the data from these different conditions, mosaicism of aberrant methylation suggests that the epigenetic error occurs after fertilisation and affects the maintaining of the methylation signals at imprinted loci. Methylation patterns are largely erased in primordial germ cells and are re-established in sex-specific patterns in mature male and female germ cells. The key regulators in these processes are DNA methyltransferases and methyl-binding domain proteins, but these mechanisms remain largely to be elucidated. Insights in the complexity of methylation pattern dynamics in the early embryo and the mechanisms that establish and maintain genomic methylation patterns have been provided by Howell et al. [54]: In *DNA methyltransferase-1 *(*Dnmt1*) deficient mice, genomic imprints were established normally in oocytes, but there was an unexpected postzygotic loss of methylation. Possibly *Dnmt1 *is required to maintain methylation patterns at imprinted loci and only during a single S phase in the early embryo. The establishment of regular imprints could be demonstrated for the first time in the offspring of *Dnmt3L^-/- ^*mice [55]: in absence of the *DNA methyltransferase 3L *(*Dnmt3L*) other factors mark individual differentially methylated regions (DMRs) alone but for an appropriate imprinting pattern at all loci a combination of all involved factors is necessary.

## Diagnostic algorithm in patients with SRS and SRS-like features

**Figure 2:**
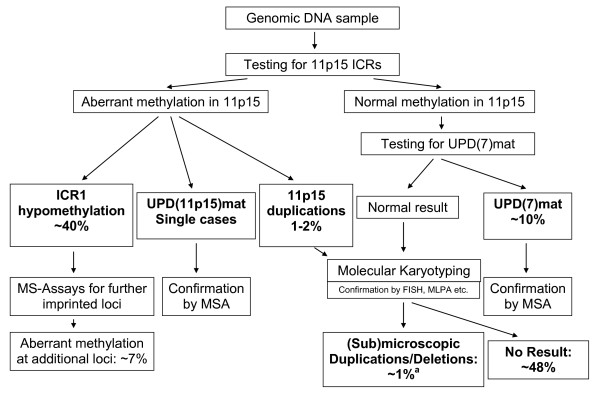
**Diagnostic algorithm in children diagnosed as SRS**. The algorithm should comprise MS-MLPA for the 11p15 loci and UPD(7)mat testing for loci on both arms of the chromosome. (^a ^the ratio is roughly estimated due to the limited numbers of systematic studies aiming on submicroscopic imbalances in SRS; MSA: microsatellite analysis; MS assays: methylation-specific assays)

With the identification of the ICR1 hypomethylation in 11p15 and the UPD(7)mat the molecular confirmation of the clinical diagnosis of SRS is now possible in ~50% of patients (fig. 2).

All currently known patients with UPD(7)mat are the result of a chromosomal nondisjunction event, in these cases the recurrence risk is not increased in the families. Meanwhile several patients with segmental UPD of the long arm of chromosome 7 have been described, thus it is meaningful to test patients for UPD(7)mat for both known imprinted loci on the short and the long arm of chromosome 7. We suggest to use methylation-specific PCR approaches for both 7p and 7q loci because they allow the detection of UPD(7)mat for diagnostic purposes and the detection of so far unknown isolated imprinting defects on chromosome 7. If a positive result is obtained microsatellite typing is indicated to confirm UPD(7)mat and to exclude the aforementioned isolated imprinting defects and deletions.

In case of prenatal testing for UPD, methylation-specific tests might be hampered by the uncertainity whether methylation is completed, thus microsatellite typing is the tool of choices.

A large group of SRS patients shows an ICR1 hypomethylation in 11p15. Several testing procedures have been reported for methylation analysis of the 11p15 loci, the advantage of the methylation-specific multiplex ligation probe-dependent analysis (MS-MLPA) approach is that copy number variation and aberrant methylation at different loci in 11p15 can be detected in one tube. Thus, methylation defects at both ICRs in 11p15 as well as duplications and UPDs of this region will be identified. However, MS-MLPA as well as the other reported tests have limitations such as sequence variants which affect the probe hybridisation or incomplete bisulphite conversion. Furthermore, we have to bear in mind that routine diagnostics is based on lymphocytes and that nearly all SRS patients with ICR1 hypomethylation are mosaics. Thus we assume that a subgroup of patients escapes molecular diagnosis because their mosaicism affects tissues other than blood cells. In case of a strong clinical suspicion of SRS but exclusion of the major (epi)genetic disturbances we therefore suggest to analyse a second cell system e.g. buccal epithelium. Whereas the MS-MLPA patterns for aberrant methylation are unambiguous and generally do not need confirmation by a second test, duplications/deletions and UPDs can only be verified by microsatellite typing with 11p15 markers or qPCR. It is currently difficult to estimate whether methylation-specific tests for 11p15 imprinting regions are an adequate tool for prenatal testing due to the uncertainty of the timing of methylation at specific loci in the embryo.

Recently, multiple hypomethylations at other loci than the ICR1 have been demonstrated in up to 7% of patients [53]. Currently, there are no obvious clinical differences between patients with isolated ICR1 hypomethylation and those with multiple imprinting defects. For research purposes, patients with ICR1 demethylation might thus be tested for further imprinted loci.

After exclusion of 11p15 epimutation and UPD(7)mat, molecular karyotyping can help to identify submicroscopic imbalances. Indeed, the frequency of chromosomal imbalances in SRS is unknown but based on two studies [10,11] on this subject we estimate that this aberration accounts for ~1% of SRS patients.

By summarising the molecular genetic data from routine diagnostic cases referred as SRS we recently showed that 11p15 epimutation and UPD(7)mat carriers do not always exhibit the unambiguous SRS phenotype [1,34]. Indeed, genetic testing for both aberrations should also be considered in case of "SRS-like" phenotypes, e.g. mild intrauterine and postnatal growth retardation (> -2SD) associated with a prominent forehead and triangular face or asymmetry as the only clinical signs. In particular, the lack of IUGR in patients with a "SRS-like" phenotype should not automatically result in exclusion from molecular testing.

Finally, the molecular proof of SRS is of particular importance considering the subjectiveness of the clinical diagnosis of SRS. With respect to genetic counselling, the identification of ICR1 hypomethylation or maternal UPD7 allows delineation of a low recurrence risk due to their de-novo occurrence. First clinical characterisations suggest that the phenotype of maternal UPD7 carriers is generally milder whereas 11p15 epimutation carriers usually present the typical picture of SRS [56,57]. Further phenotype analyses will help to find out whether the molecular subgroups of SRS respond differently to growth hormone treatment as suggested by Binder et al. [58].

## Competing interests

The authors declare that they have no competing interests.

## Authors' contributions

TE wrote the draft of the manuscript. All authors discussed, read and approved the manuscript.
